# Vibration-based gearbox fault diagnosis using a multi-scale convolutional neural network with depth-wise feature concatenation

**DOI:** 10.1371/journal.pone.0324905

**Published:** 2025-07-07

**Authors:** Van-Trang Nguyen, Quoc Bao Diep

**Affiliations:** 1 Faculty of Vehicle and Energy Engineering, Ho Chi Minh City University of Technology and Education, Ho Chi Minh City, Vietnam; 2 Computational Science and Applications Research Group, Faculty of Mechanical - Electrical and Computer Engineering, School of Technology, Van Lang University, Ho Chi Minh City, Vietnam; The University of British Columbia, AUSTRALIA

## Abstract

This article proposes a novel approach for vibration-based gearbox fault diagnosis using a multi-scale convolutional neural network with depth-wise feature concatenation named MixNet. In industrial environments where equipment reliability directly impacts productivity, safety, and operational efficiency, timely and accurate fault detection in gearboxes is of paramount importance. As critical components in manufacturing, energy production, transportation, and heavy machinery, gearboxes constitute major potential failure points, with malfunctions leading to costly downtime and, in severe cases, catastrophic incidents. The proposed method addresses these industrial challenges by integrating advanced signal processing techniques with deep learning architectures to enhance diagnostic accuracy and robustness. Specifically, MixNet utilizes multi-scale convolutional layers combined with depth-wise feature concatenation to extract discriminative features from spectrogram representations of vibration signals, generated via the Short-time Fourier transform (STFT). This approach offers several practical advantages for engineering applications, including non-invasive monitoring that eliminates the need for disassembly, early fault detection that facilitates condition-based maintenance strategies, automated diagnosis that minimizes reliance on domain-specific expertise, and robust performance under noisy and variable operating conditions. Experimental results on the Gearbox fault diagnosis dataset demonstrate that MixNet outperforms existing deep learning models, achieving a significantly higher accuracy of 99.32% with a relatively fast training time of only 4 minutes and 29 seconds. The combination of high accuracy and computational efficiency renders the proposed method well-suited for deployment in real-time monitoring systems across manufacturing plants, power generation facilities, and automotive applications, where it has the potential to reduce maintenance costs by up to 30% and improve equipment availability by enabling the detection of incipient faults before catastrophic failures.

## 1 Introduction

In the era of Industry 4.0, the stability and reliability of machinery systems play a crucial role in many industries, such as manufacturing, transportation, and energy production. Equipment failures and malfunctions cause production disruptions, pose significant safety risks, and incur high repair costs [1]. Therefore, predictive maintenance has become an increasingly important research area aimed at the early detection of abnormal signs and developing appropriate maintenance plans [2, 3]. Predictive maintenance can help minimize downtime, reduce maintenance costs, and improve overall system safety and efficiency by identifying potential faults before they escalate into severe failures.

Despite significant advances in predictive maintenance technologies, accurate fault diagnosis of gearboxes remains a significant challenge in industrial applications. Traditional diagnostic approaches often exhibit several limitations, including low sensitivity to early-stage faults, susceptibility to environmental noise, and reliance on expert knowledge for manual feature extraction and interpretation. These shortcomings may result in missed fault detections or false alarms, potentially leading to unexpected equipment failures or unnecessary maintenance actions. According to recent industry reports, gearbox failures contribute to approximately 30% of all mechanical system breakdowns in manufacturing facilities, with an average downtime cost exceeding 5,000 USD per hour in medium to large-scale operations. Moreover, the increasing complexity of modern industrial systems, characterized by diverse and dynamic operating conditions, along with the growing demand for autonomous monitoring solutions, underscores the need for more robust and accurate diagnostic techniques. To address these challenges, this study proposes an advanced deep-learning architecture specifically designed to overcome the limitations of conventional gearbox fault diagnosis methods, offering improved accuracy, adaptability, and robustness under real-world industrial conditions.

Among various predictive maintenance techniques, vibration analysis has emerged as a highly effective method for monitoring the condition of rotating machinery, particularly gearboxes. As critical components in numerous industrial systems, gearbox failures can result in significant operational downtime and, in severe cases, catastrophic outcomes. The fundamental premise of vibration analysis lies in the fact that mechanical faults such as those in gears and bearings induce abnormal vibration patterns, which can be detected using vibration sensors [4, 5]. By analyzing these vibration signals, it is possible to assess the health status of internal components and identify emerging faults at an early stage.

In recent years, the rapid advancement of artificial intelligence (AI) and machine learning techniques has brought new opportunities for vibration-based fault diagnosis [6–8]. In particular, the combination of time-frequency representations, such as spectrograms, and deep learning models, especially convolutional neural networks (CNNs), has shown great promise [9]. Spectrograms visually represent vibration signals in the time-frequency domain, allowing for observing energy distribution across different frequency bands over time. Meanwhile, CNNs are a powerful class of deep learning models that are powerful at automatically extracting relevant features from images and learning complex patterns. By treating spectrograms as input images and applying CNNs for analysis, researchers have developed automated systems that can detect anomalies and accurately diagnose various types of gearbox faults.

The current state of research in vibration-based gearbox fault diagnosis has progressed substantially with the emergence of deep learning techniques. Recent studies have explored various deep learning architectures, including basic convolutional neural networks (CNNs), recurrent neural networks (RNNs), and hybrid models combining multiple neural network types. While these approaches have demonstrated improvements over traditional methods, they face persistent challenges in extracting discriminative features across different operational conditions, efficiently processing multi-sensor data, and maintaining robustness against noise interference. Several studies have attempted to address these limitations through transfer learning, attention mechanisms, and ensemble methods, but the balance between diagnostic accuracy, computational efficiency, and generalization capability remains elusive. Furthermore, most existing approaches treat feature extraction as a single-scale process, potentially missing important patterns that manifest at different resolutions in the vibration signals. This research gap motivates our proposed multi-scale approach with depth-wise feature concatenation, which aims to capture complementary information across different scales while maintaining computational efficiency.

Despite significant advances, several critical research gaps persist in vibration-based gearbox fault diagnosis. Current approaches often treat feature extraction as a single-scale process, potentially missing patterns that manifest at different signal resolutions. Efficient feature fusion across scales remains challenging, with many methods struggling to balance high accuracy with computational efficiency for real-time industrial deployment. Moreover, most existing models exhibit reduced performance under varying operational conditions. Recent advancements in digital twin methodologies, as demonstrated by [10] on gear wear monitoring and neuro-fuzzy guided diagnostic frameworks, such as [11] cross-modal zero-sample approach using thermography and acoustic data offer promising directions, but have yet to be fully integrated with efficient deep learning architectures for vibration analysis.

The main contribution of this article is to propose a novel approach that leverages the strengths of multi-scale convolutional neural networks and depth-wise feature concatenation for enhanced gearbox fault diagnosis. Our method involves critical steps: First, we apply advanced signal processing techniques, such as the Fourier transform, to change raw vibration signals into high-quality spectrograms. These spectrograms serve as informative input features that represent the time-frequency characteristics of the vibration signals. Next, we design a custom CNN architecture incorporating multi-scale convolutional layers and depth-wise feature concatenation. The multi-scale design allows the network to capture both local and global patterns in the spectrograms, and the depth-wise feature concatenation enables effective fusion of features from different scales. By training this CNN model on a diverse dataset of gearbox vibration signals, we obtain a powerful fault diagnosis system that can automatically identify various types of gearbox faults with high accuracy and robustness.

The proposed approach offers several significant advantages over traditional methods:

- Non-invasive monitoring: Our vibration-based method allows for the monitoring of gearbox health without the need for disassembly or direct access to internal components. This non-invasive nature makes it highly practical and cost-effective for real-world applications.
- Early fault detection: By analyzing vibration signals in the time-frequency domain using advanced MixNet models, our approach can detect slight abnormalities and incipient faults that may not be apparent in the raw time-domain signals. This early detection capability is crucial for preventing further damage and scheduling timely maintenance.
- Automated and efficient diagnosis: Once trained, our MixNet model can automatically diagnose gearbox faults based on input spectrograms without requiring manual feature engineering or expert interpretation. This automation dramatically improves the efficiency and scalability of the fault diagnosis process, making it suitable for large-scale industrial applications.
- Robustness to noise and variations: The multi-scale architecture and depth-wise feature concatenation in our MixNet model enhance its ability to capture robust and discriminative features from spectrograms, even in noise or variations in operating conditions. This robustness is essential for reliable fault diagnosis in real-world scenarios.

The remainder of this article is organized as follows: Sect 2 provides an overview of related work in the field of vibration-based gearbox fault diagnosis, highlighting the recent advances and challenges. Sect 3 presents the proposed methodology, including the signal processing techniques, the multi-scale CNN architecture, and the depth-wise feature concatenation mechanism. Sect 4 describes the experimental setup, including the dataset, evaluation metrics, and baseline methods used for comparison. Sect 5 presents and discusses the experimental results, demonstrating the effectiveness and superiority of the proposed approach. Finally, Sect 6 concludes the article and outlines future research directions.

## 2 Related work

In recent years, vibration-based gearbox fault diagnosis has attracted significant attention from the research community. The combination of signal representation in image form (such as spectrograms) and deep learning models (especially convolutional neural networks) has opened up many new approaches. This section will provide an overview of recent representative studies in this field, highlighting significant contributions and remaining challenges, thereby emphasizing the motivation and direction of our proposed research.

Digital twin technology has emerged as a powerful methodology for machinery health management, creating virtual replicas of physical systems to model degradation processes. Feng *et al*. [12] developed a digital twin-driven approach for intelligent assessment of gear surface degradation that combines high-fidelity digital models with transfer learning to accurately monitor wear progression under different operating conditions. Similarly, Zhang *et al*. [13] proposed a digital twin-driven partial domain adaptation network for rolling bearing fault diagnosis, which utilizes simulation data to overcome the scarcity of real-world fault data through a Transformer-based network with selective adversarial training. The integration of domain adaptation techniques with fault diagnosis has also shown significant promise. Zhang *et al*. [14] introduced a supervised contrastive learning-based domain adaptation network that achieves cross-domain fault diagnosis by simultaneously implementing domain-level alignment and class-level alignment through contrastive learning. These approaches represent the frontier of intelligent fault diagnosis by addressing key challenges such as limited training data, domain shifts between simulation and real-world environments, and the need for interpretable results.

Deveci *et al*. [15] conducted a comprehensive study comparing various CNN models and methods for transforming one-dimensional vibration signals into two-dimensional images to detect bearing faults. The study utilized 18 different image representations, such as spectrogram, CWT, and scattergram, as inputs to four CNN architectures: GoogLeNet, ResNet-50, SqueezeNet, and Inception-ResNet-v2. The CWRU Bearing dataset was used for this research. The best result achieved was an accuracy of 99.89% using the Scattergram Filter Bank 1 image as input and the ResNet-50 network. Notably, 10 out of the 72 methods investigated yielded accuracies above 99.5%. The study is one of the most comprehensive investigations of the bearing fault classification problem, with 72 different methods examined. The authors employed image transformation methods rarely used before, such as scattergram, and achieved the best results with this approach. The research provides valuable insights into which signal-to-image transformation methods can extract more distinct features and which CNN architectures better discriminate between different fault types. Overall, this study serves as a valuable reference when summarizing related works on the application of transfer learning and CNNs for bearing fault detection based on image representations of one-dimensional signals.

Dong *et al*. [16] addressed the challenge of gearbox fault diagnosis in imbalanced datasets, where the number of healthy samples significantly outweighs the number of faulty samples. To improve the accuracy of defect recognition, they suggested a unique Dynamic Normalization Supervised Contrastive Network (DNSCN) with a multiscale compound attention mechanism. In order to efficiently mine features from the signals, DNSCN uses a multiscale adaptive feature extractor (MAFE). A multiscale compound attention mechanism (MCAM) then reweights the features to increase diagnostic accuracy. They devised a dynamic normalized supervised contrastive loss function that balances the contribution of minority and hard-to-classify data in order to address class imbalance. Using two gearbox datasets with exceptional imbalance ratios (up to 40:1), DNSCN outperformed other approaches such as SVM, MA1DCNN, ODC-Net, SupCon, SMOTE, under-sampling, reweighting, and WGAN-GP, achieving accuracies of 91.58% and 90.96%, respectively. The study highlighted the effectiveness of DNSCN in handling class imbalance and improving fault diagnosis accuracy. However, the authors acknowledged limitations in recognizing unknown fault classes and the need for further interpretability of the feature extractor. They suggested future research to incorporate incremental learning and physical degradation processes for more practical fault diagnosis.

Jiang *et al*. [17] addressed the challenge of gearbox fault diagnosis in scenarios where limited real-world data is available. They proposed an enhanced unsupervised domain adaptation method that combines a vibration response mechanism with a domain mapping technique. The vibration response mechanism was used to generate labeled simulation signals with clear physical meaning, addressing the lack of real-world fault data. To bridge the gap between simulation and experimental data, a domain mapping method was introduced to align their distributions, improving diagnostic accuracy and speed. This domain mapping approach can be easily integrated into existing unsupervised domain adaptation models without requiring structural modifications. Experiments on two gearbox datasets showed that the proposed method achieved high diagnostic accuracy (above 95%) with a limited number of experimental samples, outperforming existing domain adaptation models. The study highlighted the potential of using simulation data combined with domain mapping to improve fault diagnosis in scenarios with limited real-world data. However, the authors acknowledged the need for further research to develop more accurate simulation models that can eliminate the need for experimental data altogether.

Zhi *et al*. [18] addressed the limitations of traditional gearbox fault detection methods that rely on comparing sideband frequencies in the frequency domain. They proposed a novel approach based on meshing frequency modulation (MFM) analysis, which directly identifies localized gear faults by analyzing the changes in MFM areas between healthy and faulty conditions. The method involves decomposing the vibration signal into multiple frequency bands and constructing an MFM index to identify the MFM region. A new MFM area emerges in the faulty condition, indicating the presence of a localized fault. The effectiveness of the proposed method was demonstrated using both synthetic and experimental data from a gearbox test rig. The study showed that the proposed method successfully detected localized faults like spalling and chipping, achieving a 0% collision rate and 100% target reachability in simulations. However, the authors acknowledged that the method has limitations in detecting weak gear faults, which presents an area for future research.

Wang *et al*. [19] aimed to improve the accuracy and efficiency of gearbox fault detection using deep learning. They proposed a method that integrates the convolutional block attention module (CBAM) into the ResNeXt50 network. The CBAM enhances feature extraction by emphasizing important channels and spatial features in the images generated from vibration signals. They compared the performance of CWT and STFT for converting one-dimensional vibration signals into two-dimensional images, finding that CWT yielded better results. The proposed CBAM-ResNeXt50 model achieved high accuracy (99.95% and 99.875%) and faster convergence speed compared to other convolutional neural networks like DenseNet121, ResNeXt50, ResNet50, and AlexNet. The study demonstrated the effectiveness of CBAM in improving fault classification accuracy and reducing training time. However, the authors acknowledged the limitation of limited data availability for training deep learning models and suggested future research to address this challenge.

Yu *et al*. [20] addressed the challenge of accurately diagnosing gearbox faults in planetary gear systems, particularly when dealing with minor faults that are difficult to identify using traditional methods. They proposed a novel approach that combines a digital twin model with a data-driven method for signal fusion. They first constructed a digital twin of the planetary gearbox using Hertz contact theory and extended finite element methods, enabling dynamic monitoring and simulation. They then extracted features from both real and simulated vibration signals, matching them based on correlation coefficients. Finally, they fused the real and simulated signals using variational mode decomposition (VMD) and phase correction, enhancing fault features. Experimental results showed that the virtual-real fusion method effectively improved the performance of traditional signal diagnosis methods, achieving accurate fault diagnosis with improved clarity of fault features. The study highlighted the potential of digital twin-based signal fusion for gearbox fault diagnosis, particularly in scenarios with minor faults. However, the authors acknowledged that further research is needed to address the limitations of the simulation model in accurately representing real-world conditions and to explore the application of the proposed method in more complex and dynamic scenarios.

Karabacak *et al*. [21] investigated the application of vibration, sound, and thermal aspects for intelligent problem diagnosis of worm gearboxes under varied operating situations. They created a test rig that mimics real-world operational situations by gathering data at various loading rates and speeds. For the purpose of defect identification and classification, they employed both ANN and SVM classifiers after extracting features from the temporal and frequency domains of the gathered data. Combining information from vibration, sound, and thermal imaging produced the best classification performance, according to the study, with ANN and SVM reaching 99.2% and 98.7% accuracy for defect detection, respectively. The research also demonstrated that the combination of thermal imaging characteristics and sound produced superior outcomes compared to the use of vibration alone. The authors highlighted the importance of selecting appropriate features and using multiple data sources for improved fault diagnosis. However, the study was limited to a single gearbox type and did not investigate the performance of the proposed method in more complex scenarios with multiple gearboxes or dynamic environments.

Guo *et al*. [22] addressed the limitations of existing data-driven fault detection methods for gearboxes, which primarily focus on single-sensor data and struggle to accurately capture fault features. They put out a brand-new feature fusion framework for multi-sensor driven gearbox intelligent defect detection that is based on multiscale cyclic frequency demodulation (MCFD). The limitations of conventional cyclic spectrum analysis are overcome by the MCFD approach, which efficiently demodulates fault modulation features from vibration signals. In order to create a multi-sensor information fusion covariance matrix (MIFCM), the framework further makes use of grey correlation analysis to fuse information from numerous sensors. The MCFD-based feature fusion framework performed better than other feature fusion systems like LLE, mRMR, and t-SNE, delivering higher diagnostic accuracy, according to experimental results on helical and planetary gearboxes. For example, in the planetary gearbox case, the MIFCM achieved an average diagnosis accuracy of 98.21% for the normal condition, 95.37% for sun gear chipping, 96.22% for sun gear misalignment, and 96.56% for sun gear with 0.7 mm misalignment. The study also showed that the MIFCM maintained high accuracy even with fewer training samples. The authors acknowledged that the proposed framework may have limitations in handling noisier environments and scenarios with fewer samples, suggesting further research to address these challenges.

Kumar *et al*. [23] studied the use of higher-order moments (HOM) for gearbox failure diagnostics, presenting a new feature called LHOM (logarithmic amplitude of HOM). They looked at how LHOM characteristics behaved under various localized fault conditions and operating settings. They discovered that LHOM was closely correlated with fault severity and showed a linear trend with the order of moments beyond the fifth order. Using three feature selection methods (PCC, NCA, and Relief-F) and five well-known ML classifiers (MCSVM, KNN, DT, RF, and NB), they evaluated the effectiveness of LHOM features against traditional time-domain features on three benchmark gearbox datasets. The findings demonstrated that LHOM features significantly improved classification accuracy over traditional features, especially when combined with the MCSVM classifier. For instance, the MCSVM classifier increased accuracy by an average of 8.09% at PCC, 4.69% at NCA, and 10.11% at RF. The study highlighted the potential of LHOM features for gearbox fault diagnosis, particularly in scenarios with localized faults. However, the authors acknowledged that further research is needed to investigate the effectiveness of LHOM features in more complex scenarios with multiple fault types and to explore the application of deep learning methods for fault diagnosis.

Ha and Fink [24] addressed the problem of cross-domain defect diagnostics for planetary gearboxes in situations when only healthy data is available in the target domain. Using the Health Data Map (HDMap) representation of vibration signals, they proposed two unique domain knowledge-informed data synthesis methods: Scaled CutPaste and FaultPaste. Scaled CutPaste synthesizes faulty samples by pasting a scaled fault signature onto healthy samples in the target domain, while FaultPaste extracts fault signatures from the source domain using an autoencoder and pastes them onto healthy target domain samples. The study demonstrated the effectiveness of these methods in accurately diagnosing faults and estimating fault severity levels, even in cases of extreme domain shifts. For example, the FaultPaste method achieved an average accuracy of 99.4% for fault detection and an average ROC-AUC score of 0.999 for fault severity prediction across 12 domain shift tasks. The authors highlighted the potential of these methods for improving fault diagnosis performance in real-world applications where only healthy data is available in the target domain. However, the study acknowledged that further research is needed to explore the performance of the proposed methods in more complex scenarios with multiple fault types and to investigate the impact of different operating conditions on the fault signature pattern.

Through the review of related studies, the application of CNNs and image-based signal representations has achieved promising results in gearbox fault diagnosis. However, some challenges remain to be addressed, such as accuracy, effective transfer learning, and improving model interpretability. This study proposes a novel approach based on a multi-scale and depth-wise feature concatenation to overcome these limitations. The following section will present our proposed method in detail.

## 3 The proposed methodology

An automatic machinery vibration analysis solution based on the combination of spectrogram images and the multi-scale convolutional neural network with depth-wise feature concatenation named MixNet is proposed in this study. The process consists of two main steps: (1) analyzing and transforming vibration signals into spectrogram images and (2) using the proposed MixNet to analyze the spectrograms for detecting anomalies and diagnosing faults. The following subsections will present details about each step in this process.

### 3.1 Vibration analysis and transformation

Transforming vibration waveform signals into spectrogram images is a technique that provides a more visual perspective on vibration data. Specifically:

- Vibrations are waveform signals in the time domain. They represent the intensity and frequency of vibrations over time.
- A spectrogram is an image containing time, frequency, and amplitude where:
  - The horizontal axis represents time;
  - The vertical axis represents frequency;
  - The color or brightness of pixels represents the amplitude (or energy) at each corresponding frequency.
- The Short-time Fourier transform (STFT) algorithm is commonly used to transform vibration waveform signals into spectrograms. This algorithm divides the signal into short time frames and applies the Fourier transform to each frame to analyze the frequency components.
- Representation using spectrograms allows us to visualize the distribution of vibration energy across different frequencies over time, enabling intuitive analysis of vibration characteristics.

Assuming the vibration signal is *x*(*t*), the STFT is defined as in Eq 1.

$$X(\tau ,f)=\int x(t)w(t-\tau )e^{-j2\pi ft}dt$$ (1)

where:

- X(τ,f) is the STFT of the signal *x*(*t*),
- τ is the start time of each time frame,
- *f* is the frequency,
- *w*(*t*) is the window function to extract each time frame, usually the Hann or Hamming function,
- *j* is the imaginary unit, *j*^2^ = −1.

The window function *w*(*t*) can be defined as in Eq 2 (for example, with the Hann window).

$$w(t)=\left\{ \begin{array}{cc} (1-\cos(2\pi t/T))/2 & \text{if }0\leq t\leq T\;\\ 0 & \text{if }t<0\text{ or }t>T \end{array}\right.$$ (2)

Where *T* is the length of each time frame.

After calculating the STFT, we obtain a complex matrix X(τ,f), the real and imaginary parts represent the amplitude and phase of each frequency component at each time point, respectively. The spectrogram S(τ,f) is calculated by taking the absolute value of X(τ,f), as in Eq 3.

S(τ,f)=|X(τ,f)| (3)

The spectrogram S(τ,f) is a real matrix that can be represented as an image, where the horizontal axis represents time τ, the vertical axis represents the frequency *f*, and the value at each pixel represents the color intensity corresponding to the amplitude S(τ,f).

**Fig 1:**
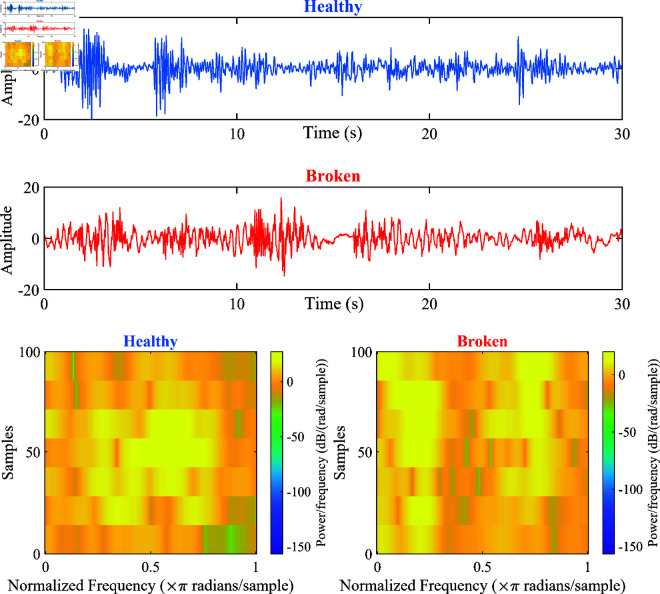
Waveforms and spectrograms of vibration signals from healthy and broken gearboxes.

In this study, Fig 1 represents vibration signals from a healthy gearbox (blue) and a broken gearbox (red). In the spectrogram, the horizontal axis (Normalized Frequency) represents the normalized frequency in units of radians/sample. The vertical axis (Samples) represents the index of signal samples over time. Each sample corresponds to a specific point in time in the original signal. This axis indicates the distribution of signal energy over time. The color (Power/frequency) represents the power spectral density (PSD) of the signal at each point (frequency, time), with units of dB/(radians/sample). PSD indicates the average energy level of the signal at each frequency and corresponding time, where brighter colors indicate higher PSD and darker colors indicate lower PSD.

### 3.2 The multi-scale convolutional neural network with depth-wise feature concatenation

We employ the multi-scale convolutional neural network with depth-wise feature concatenation architecture (MixNet) consisting of multiple convolutional and fully connected layers to analyze the spectrograms. The convolutional layers help extract local features from the input images, while the fully connected layers perform classification based on those features.

**Fig 2:**
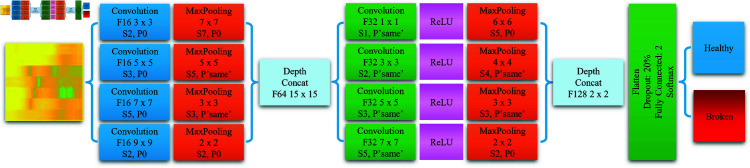
The multi-scale convolutional neural network with depth-wise feature concatenation architecture.

Fig 2 illustrates the proposed MixNet architecture, detailing the parameters of each layer. Specifically, the notation “F" represents the number of filters (or kernels) in each convolutional layer, “S" denotes the stride value, and “P" indicates the padding value. For example, “F16 3×3 S2, P0" means that the convolutional layer has 16 filters with a size of 3×3, uses a stride of [2 2], and padding of [0 0 0 0].

The proposed MixNet architecture for classifying spectrogram images consists of two parallel branches, each containing a series of convolutional layers and max pooling layers.

The first branch includes 4 convolutional layers with 16 filters each and kernel sizes of 3×3, 5×5, 7×7, and 9×9, respectively. A max pooling layer with different sizes and strides follows each convolutional layer. These pooling layers help reduce the size of the feature maps and increase invariance to object translation and scaling.

The second branch has a similar structure, comprising 4 convolutional layers with 32 filters each and a ReLU activation layer following each convolutional layer. At each branch’s end is a series of max pooling layers to reduce the spatial dimensions. Following the multi-scale feature extraction through these parallel branches, their outputs are concatenated along the depth dimension, forming a 64-channel feature with a size of 15×15 and a 128-channel feature with a size of 2×2. This depth-wise concatenation effectively combines features extracted at different scales, enabling the network to simultaneously leverage both fine-grained details and global context information.

Finally, these features are passed through a flattened layer to convert them into a 1-dimensional vector, a 20% Dropout layer to reduce overfitting, and a fully connected layer to classify the input into the Healthy or Broken class.

By utilizing convolutional layers with different kernel sizes and a two-branch structure, this architecture can learn features at various scales of the input image, thereby increasing the accuracy of the spectrogram image classification task.

## 4 Experimental setup

### 4.1 Vibration signal data

This study uses the “Gearbox Fault Diagnosis” data set obtained from the Open Energy Data Initiative (OEDI) open data repository and provided by the authors in the original publication [25]. The data set consists of vibration recordings collected by four vibration sensors placed in four different directions on “SpectraQuest’s Gearbox Prognostics Simulator” device.

Data is recorded in two situations: 1) Gearbox operates normally, and 2) Gearbox has damaged teeth. The load varies from 0% to 90% for each situation, divided into 10 steps. There are 20 comma-separated values data files, of which 10 correspond to gearboxes that operate normally and 10 correspond to gearboxes with broken teeth. Each file is a record corresponding to the load level from 0% to 90%.

**Fig 3:**
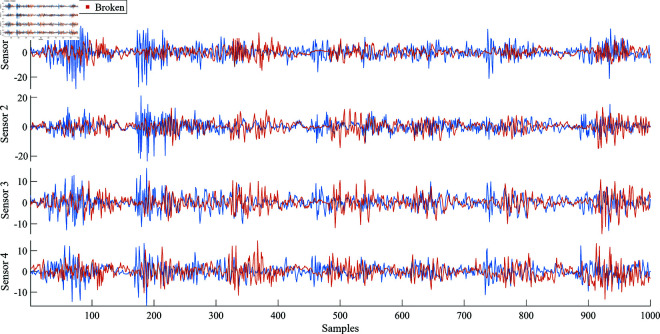
Comparison of vibration signals from four sensors in healthy and broken gearbox states.

Fig 3 presents vibration signals collected from four different sensors, comparing a gearbox’s healthy and broken states. The figure illustrates 1.000 samples for each sensor, with amplitudes ranging from approximately -20 to 20 for Sensors 1 and 2 and -10 to 10 for Sensors 3 and 4. An indistinction can be observed between the healthy and broken signals across all sensors, indicating the challenges of vibration signal analysis for gearbox fault detection by the time-domain signals.

These time-domain signals can be transformed into spectrogram images using the Short-time Fourier transform, as described in our methodology and the following subsection. The MixNet architecture can then be applied to extract and combine features from these spectrograms at various scales. The model’s ability to distinguish between healthy and broken patterns can be evaluated using these signals, demonstrating its effectiveness in real-world gearbox fault diagnosis scenarios.

### 4.2 White Gaussian noise injection

To evaluate the robustness of our proposed model, we introduced additive white Gaussian noise (AWGN) to the raw vibration signals. This process increases the presence of environmental noise and measurement uncertainties commonly encountered in real-world applications. The AWGN was added using Eq 4, which adds white Gaussian noise to the input signal, where X represents the original vibration signal, and Y is the resulting noisy signal. The second parameter (SNR), set to 20, specifies the signal-to-noise ratio in decibels. This setting corresponds to a moderate noise level, providing a challenging yet realistic scenario for our fault diagnosis system.

Y = AWGN(X, SNR) (4)

The AWGN part is particularly relevant in our context as it approximates unpredictable factors, such as the thermal noise in electronic systems and other random processes that affect signal acquisition. By setting the SNR to 20, we maintain a balance where the original signal characteristics are preserved while introducing sufficient noise to test the model’s resilience. This approach allows us to assess the performance of our fault diagnosis techniques under broader conditions that more closely resemble those encountered in industrial settings, where clean, noise-free signals are rarely available.

### 4.3 Transformed data

To create the transformed data for the MixNet model, the raw vibration signals are transformed into spectrogram images using the STFT technique. The STFT is applied to each vibration signal with the following parameters:

- Window function: Hamming window
- Window length: $n_{sc}=\lfloor N_{x}/4.5\rfloor =\lfloor 1024/4.5\rfloor =227$ samples
- Hop length (overlap between windows): 50% overlap, i.e., 113 samples
- Number of FFT points: $\max(256,2^{\lceil \log_{2}(n_{sc})\rceil })=256$

The resulting spectrograms are then resized to a fixed size of 224×224×3 pixels to match the input size of the MixNet model. The pixel values of the spectrogram images are normalized to the range [0,1] to improve the convergence speed and stability of the training process.

The spectrogram dataset consists of 20,000 spectrogram images, with 10,000 images corresponding to healthy gearboxes and 10,000 images corresponding to broken gearboxes. The dataset is randomly divided into three subsets:

- Training set: 14,000 images (70%)
- Validation set: 2,000 images (10%)
- Testing set: 4,000 images (20%)

**Fig 4:**
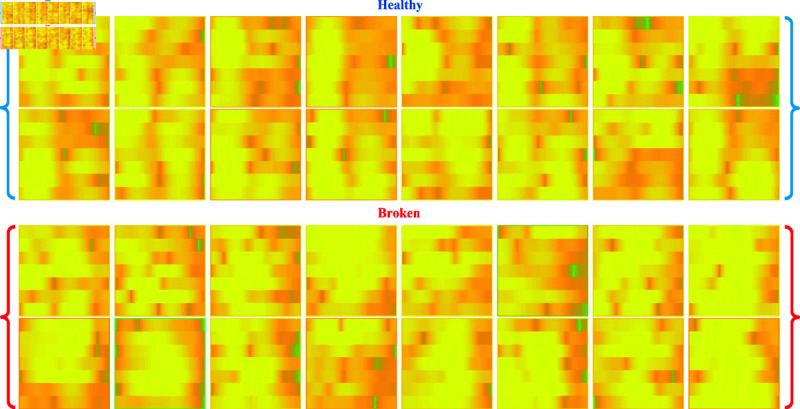
Spectrogram images generated from vibration signals of healthy and broken gearbox conditions.

Fig 4 presents the first sixteen spectrogram images for each condition derived from gearboxes’ vibration signals in healthy and broken conditions. These spectrograms were generated using the STFT applied to the raw vibration signals described earlier. The horizontal axis represents time, while the vertical axis depicts samples. The color intensity indicates the magnitude of the signal at each time-sample point, with brighter colors representing higher energy levels. These visual representations provide a more precise differentiation between healthy and broken operations, serving as valuable input for our proposed MixNet architecture to extract and analyze multi-scale features for accurate fault diagnosis.

The training set is used to train all investigated models, while the validation set is used to tune the hyperparameters and monitor the model’s performance during training to avoid overfitting. The testing set is used to evaluate the final performance of the trained model on unseen data.

### 4.4 Comparative analysis of deep learning models

To thoroughly assess the performance of the proposed network, several well-known models were selected for comparison. These models have been widely utilized in various image classification and signal processing domains. The comparative analysis focuses on evaluating critical metrics such as accuracy, precision, recall, and computational efficiency. The following models were chosen for this study:

- MLPC (a multi-sensor vibration signal fault diagnosis method under less computing resources) [26]: a specialized architecture designed for diagnosing faults in multi-sensor vibration signals. MLPC has been successfully applied in various vibration signal analysis tasks, demonstrating its effectiveness in capturing discriminative features.
- SqueezeNet [27]: a compact and efficient neural network architecture designed for image classification tasks. SqueezeNet achieves comparable accuracy to AlexNet while requiring significantly fewer parameters, making it suitable for resource-constrained environments.
- AlexNet [28]: a groundbreaking convolutional neural network that revolutionized the field of computer vision. AlexNet achieved remarkable results in the ImageNet Large Scale Visual Recognition Challenge (ILSVRC) 2012, demonstrating the potential of deep learning for image classification.
- GoogLeNet [29]: a deep and wide convolutional neural network architecture that introduced the concept of Inception modules. GoogLeNet won the ILSVRC 2014 competition, showcasing its ability to learn complex features and achieve high accuracy in image classification tasks.

The comparative analysis of MixNet against these established architectures provides valuable insights into its performance and suitability for the task at hand. By evaluating various metrics such as accuracy, precision, recall, and F1-score, the strengths and limitations of the proposed network can be thoroughly assessed, enabling informed decisions for real-world applications in gearbox fault diagnosis.

We implemented the MixNet and selected models, including MLPC, SqueezeNet, AlexNet, and GoogLeNet, using MATLAB 2024a on Windows 11 and standard PC with the specifications: GPU: NVIDIA GeForce RTX 3060 Ti with 8.0 GB dedicated memory, 64.0 GB of system memory, and a 13th Gen Intel(R) Core(TM) i7-13700KF CPU with a base speed of 3.40 GHz. Transfer learning techniques were employed to adapt AlexNet and GoogLeNet for the task of gearbox fault diagnosis using spectrogram images. The output layers were adjusted to match the number of fault classes in the dataset. By leveraging the pre-trained weights of these networks on large-scale image datasets, they can be fine-tuned to effectively classify spectrogram images for gearbox fault diagnosis.

All models were trained using the Adam optimizer [30] with an initial learning rate of 0.001, batch size of 64, and a maximum of 100 epochs. Other parameters not presented here were set as specified in the original publications.

All experiments were independently repeated 10 times to ensure the reliability and robustness of our results. This approach mitigates the potential impact of random initialization and helps avoid drawing conclusions based on chance occurrences. Using multiple runs provides a more comprehensive understanding of the model’s performance across different initializations and data splits.

Furthermore, we employed the Wilcoxon rank sum test (WRT) to statistically validate our results [31]. The WRT is a non-parametric statistical test that compares the performance of two methods across multiple runs. The WRT was chosen because it does not assume a normal data distribution and is less sensitive to outliers than parametric tests like the t-test. It helps determine whether the observed differences in performance between our proposed MixNet and the investigated models are statistically significant, thus providing a more rigorous evaluation of our method’s effectiveness.

## 5 Results and discussion

This section presents and analyzes the experimental results of the proposed gearbox fault diagnosis method based on a multi-scale convolutional neural network with depth-wise feature concatenation. The experiments were performed using the “Gearbox Fault Diagnosis" dataset from the OEDI repository, which comprises 20,000 vibration signal samples, with 10,000 samples healthy gearboxes and 10,000 corresponding to faulty ones. The raw vibration signals were converted into spectrogram images using STFT with a Hamming window, 50% overlap, and 256 FFT points. The resulting spectrograms were resized to a resolution of 224x224x3 and normalized to the [0,1] range, as described in Sects 3 and 4.

Table 1 provides a comparative evaluation of test set accuracy and training time across the examined models. The results indicate that the proposed MixNet architecture outperforms the other models in terms of accuracy, precision, recall, and F1-score. The MixNet achieves an accuracy of 99.32%, outperforming all other approaches while maintaining a relatively short training time of approximately 4 minutes and 29 seconds. Although GoogLeNet achieves a slightly higher accuracy of (99.39%), its training time is significantly longer (approximately 19 minutes and 24 seconds), nearly four times that of MixNet. SqueezeNet also demonstrates competitive performance with an accuracy of 99.22% but requires 15 minutes and 23 seconds to train, which is still longer than GoogLeNet. In contrast, AlexNet and MLPC show considerably lower performance, with accuracies of 96.35% and 93.51%, respectively. Among all models, AlexNet exhibits the longest training time, nearly 24 minutes, whereas MLPC is the second fastest in terms of training time, following MixNet.

**Table 1:** Performance and time comparison of different models on the Gearbox Fault Diagnosis dataset (mean ± std).

Model	Accuracy	Precision	Recall	F1-score	Time (mm:ss)
MixNet	99.32±0.17%	99.32±0.17%	99.32±0.17%	99.32±0.17%	04:29±00:12
MLPC	93.51±0.53%	93.51±0.53%	93.52±0.52%	93.51±0.53%	05:23±00:10
SqueezeNet	99.22±0.62%	99.22±0.62%	99.23±0.61%	99.22±0.62%	15:23±00:21
AlexNet	96.35±3.89%	96.35±3.89%	96.43±3.76%	96.35±3.89%	24:09±02:59
GoogLeNet	99.39±0.31%	99.39±0.31%	99.39±0.31%	99.39±0.31%	19:24±01:09

To ensure the clarity and reproducibility of our results, we provide formal definitions of the statistical metrics utilized in this study. Accuracy represents the ratio of correctly predicted instances to the total number of predictions and is computed as shown in Eq 5.

Accuracy = (TP + TN) / (TP + TN + FP + FN) (5)

where TP (True Positives) denotes the number of broken gearboxes correctly classified as broken, while TN (True Negatives) refers to healthy gearboxes correctly classified as healthy, FP (False Positives) corresponds to healthy gearboxes incorrectly classified as broken, and FN (False Negatives) represents broken gearboxes incorrectly classified as healthy. Precision, which quantifies the proportion of correctly identified broken gearboxes among all instances predicted as broken, is calculated as shown in Eq 6.

Precision = TP / (TP + FP) (6)

Recall (also known as sensitivity) measures the proportion of actual broken gearboxes that are correctly identified by the model. It is calculated as presented in Eq 7.

Recall = TP / (TP + FN) (7)

The F1-score provides a balanced measure of a model’s performance by combining precision and recall through their harmonic mean, as defined in Eq 8.

F1-score=2×(Precision×Recall)/(Precision+Recall) (8)

Together, these metrics offer a comprehensive assessment of model’s performance across multiple dimensions of the classification task.

Table 1 also presents a more detailed statistical analysis of model accuracy by reporting the mean and standard deviation over 10 independent runs. The results indicate that MixNet, GoogLeNet, and SqueezeNet exhibit stable classification performance, with minimal variability across runs (standard deviation less than 1%). In contrast, AlexNet demonstrates greater fluctuation, with a standard deviation reaching up to 3.89%.

To assess the statistical significance of performance differences, we employed the non-parametric Wilcoxon rank-sum test to compare the accuracy of MixNet with each of the remaining models, as summarized in Table 2. The resulting ρ-values indicate that the performance differences between MixNet and both SqueezeNet and GoogLeNet are not statistically significant (ρ>0.05), whereas the differences with AlexNet and MLPC are statistically significant (ρ<0.05), confirming the superior performance of MixNet.

**Table 2:** Statistical significance of MixNet accuracy compared to other models.

Model	Accuracy	Ranking	Hypothesis	ρ-value
MixNet	99.32±0.17%	1	-	-
GoogLeNet	99.39±0.31%	1	0	3.63e-01
SqueezeNet	99.22±0.62%	1	0	6.49e-01
AlexNet	96.35±3.89%	2	1	7.20e-03
MLPC	93.51±0.53%	3	1	1.79e-04

The proposed MixNet architecture achieves superior fault diagnosis accuracy while maintaining high computational efficiency. With only 181.1K parameters, MixNet is significantly more lightweight compared to AlexNet (61M) and GoogLeNet (6.8M), making it highly suitable for deployment on resource-constrained devices for real-time gearbox monitoring.

Demonstrating top-tier accuracy, fast training time, and exceptional stability, MixNet has proven its effectiveness in gearbox fault diagnosis based on vibration signal analysis. These findings highlight the potential for practical applications of the proposed method in industrial gearbox monitoring and maintenance systems. Moreover, the results underscore the advantage of integrating multiscale processing with depth-wise feature concatenation, which enhances the model’s capacity for feature representation and classification of vibration signal data.

**Fig 5:**
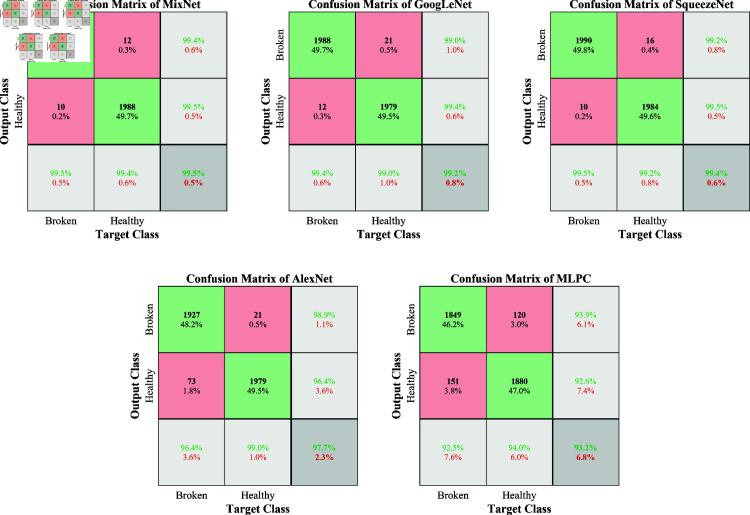
Confusion matrices for different models in gearbox fault diagnosis: MixNet, GoogLeNet, SqueezeNet, AlexNet, and MLPC, results from the first run of 10 independent runs.

Fig 5 illustrates the confusion matrices for the five evaluated models, based on the first run of the 10 independent experiments. This case study offers insight into the classification performance of each model in a specific case while acknowledging potential variability across runs.

In this specific run, MixNet exhibits strong classification performance, correctly identifying 99.5% of both healthy and faulty gearbox cases. It misclassified only 10 healthy samples (0.5%) as broken and 12 broken samples (0.6%) as healthy, indicating well-balanced performance across both classes. GoogLeNet and SqueezeNet demonstrate comparable performance, achieving overall accuracies of 99.2% and 99.4%, respectively. GoogLeNet misclassified 12 healthy samples (0.6%) and 21 broken samples (1.0%), while SqueezeNet misclassified 10 healthy samples (0.5%) and 16 broken samples (0.8%).

In contrast, AlexNet shows a noticeable drop in performance relative to the top three models, with an overall accuracy of 97.7%. It particularly underperforms in classifying healthy gearboxes, misclassifying 73 (3.6%) as broken, alongside 21 broken samples (1.0%) incorrectly predicted as healthy. MLPC demonstrates the lowest classification accuracy among the five models in this run, achieving only 93.2%. It exhibits substantial misclassifications in both classes, with 151 healthy samples (7.6%) incorrectly identified as broken and 120 broken samples (6.0%) misclassified as healthy.

While these results underscore the strong performance of MixNet in this particular run, it is important to recognize that they represent only one of the ten independent experiments. As discussed earlier, a more comprehensive evaluation of overall performance and the statistical significance of differences between models is achieved by analyzing results across all runs. Nonetheless, this case study offers a representative example of each model’s classification capability and provides valuable insight into their practical effectiveness for gearbox fault diagnosis.

**Fig 6:**
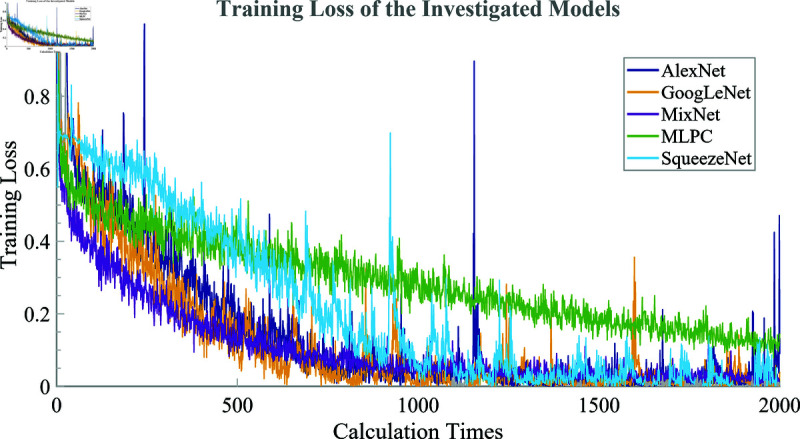
Training loss of the investigated models over calculation times.

Fig 6 presents a comparative analysis of the training loss trajectories for five models: AlexNet, GoogLeNet, MixNet, MLPC, and SqueezeNet. The graph illustrates the progression of training loss over 2000 iterations, providing valuable insights into the learning process of each model.

The most prominent feature observed in the graph is the superior performance of MixNet. It demonstrates the fastest convergence rate, reaching a low training loss within the first 500 iterations and maintaining stability afterward. This rapid convergence, combined with a consistently low overall loss, indicates that MixNet exhibits the most efficient learning capability among the models evaluated.

GoogLeNet shows the second-best performance, achieving relatively fast convergence and maintaining a stable, low training loss after approximately 1000 calculation times. SqueezeNet exhibits moderate performance, positioning between GoogLeNet and the less efficient models, AlexNet and MLPC, which show slower convergence and higher overall training loss throughout the learning process.

AlexNet and MLPC exhibit slower convergence rates and consistently higher overall training loss compared to the other models. Notably, MLPC shows noticeable fluctuations during the early stages of training, indicating a less stable and less efficient learning process relative to the deep learning-based architectures.

After approximately 1000 calculation times, most models reach a stable state, with MixNet and GoogLeNet demonstrating the highest levels of stability. Such stability is essential for ensuring reliable and consistent performance in real-world applications, particularly in dynamic industrial environments where robustness is critical.

**Fig 7:**
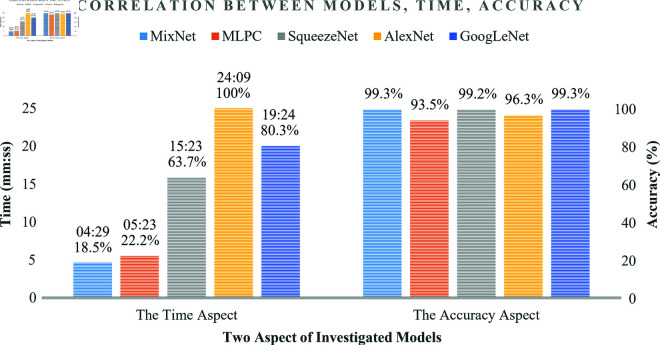
Comparison of deep learning models for gearbox fault diagnosis: Accuracy (higher is better) vs. Training Time (lower is better).

Fig 7 presents a comparative analysis of five deep learning models (MixNet, GoogLeNet, SqueezeNet, AlexNet, and MLPC) in terms of classification accuracy and training time for gearbox fault diagnosis. The graph effectively illustrates the trade-off between computational efficiency and classification performance, providing insights into the practical applicability of each model in real-world scenarios.

Regarding accuracy, MixNet achieves the top-tier highest performance at 99.3%, closely followed by SqueezeNet (99.2%) and GoogLeNet (99.3%). These three models form a high-performance group with minimal differences in accuracy. AlexNet shows a slight decrease in accuracy at 96.3%, while MLPC demonstrates the lowest accuracy at 93.5%.

Regarding training time, MixNet stands out as the most efficient, requiring only 4 minutes and 29 seconds (18.5% of AlexNet’s training time). MLPC follows with 5 minutes and 23 seconds (22.2%), then SqueezeNet with 15 minutes and 23 seconds (63.7%). GoogLeNet takes 19 minutes and 24 seconds (80.3%), while AlexNet requires the longest training time at 24 minutes and 9 seconds (100%).

This visualization reveals an interesting pattern: MixNet achieves both the highest classification accuracy and the shortest training time, highlighting its exceptional efficiency and overall performance. SqueezeNet offers good balance, reaching top-tier accuracy with moderate training time. In contrast, GoogLeNet achieves intermediate accuracy but requires a longer training time compared to SqueezeNet, indicating a less optimal trade-off between performance and computational efficiency.

These findings highlight the importance of evaluating both classification accuracy and computational efficiency when selecting an appropriate model for gearbox fault diagnosis. MixNet’s superior accuracy and efficiency make it an excellent choice for most scenarios. However, the choice between these models ultimately depends on the specific requirements and constraints of the implemented fault diagnosis system, balancing the need for high accuracy with practical considerations of training time and computational resources.

## 6 Conclusions

This study proposes an effective approach to vibration-based gearbox fault diagnosis utilizing a multi-scale convolutional neural network with depth-wise feature concatenation, named MixNet. The proposed method integrates advanced signal processing techniques with deep learning architectures to achieve high diagnostic accuracy and robustness, addressing the challenges associated with fault detection in complex industrial environments.

The experimental results demonstrate the effectiveness of the proposed MixNet model, which achieves a significantly higher classification accuracy of 99.32% compared to several well-established models, including MLPC, SqueezeNet, AlexNet, and GoogLeNet. Furthermore, MixNet demonstrates relatively fast training times and consistently high stability across multiple runs. The superior performance can be attributed to the multi-scale architecture, which enables the extraction of both local and global patterns from spectrogram representations, and the depth-wise feature concatenation, which facilitates the efficient fusion of features across different scales.

The proposed method offers several distinct advantages over conventional fault diagnosis techniques:

- Non-invasive monitoring: MixNet facilitates gearbox health assessment without the need for disassembly or direct access to internal components, thereby minimizing operational disruption and maintenance costs.
- Early fault detection: The model can detect subtle abnormalities and incipient faults at an early stage, thereby preventing further degradation and supporting timely, condition-based maintenance interventions.
- Automated and efficient diagnosis: MixNet enables fully automated fault diagnosis, eliminating the need for manual feature extraction and expert interpretation, thereby enhancing diagnostic efficiency and reducing human intervention.
- Robustness to noise and operational variations: The multi-scale architecture, combined with depth-wise feature concatenation, enhances the model’s resilience to signal noise and variations in operating conditions, ensuring reliable performance in complex industrial environments.

Although MixNet demonstrates promising results, future research should aim to address several limitations, including:

- Expanding the dataset: Incorporating a larger and more diverse dataset that encompasses various fault types and operating conditions to enhance the model’s generalization capability and applicability to real-world scenarios.
- Investigating additional fault types: Assessing the capability of MixNet to detect a broader range of gearbox faults, such as bearing defects and gear misalignment, to enhance its diagnostic comprehensiveness.
- Real-time implementation: Exploring the feasibility of implementing MixNet in real-time monitoring systems to enable continuous and automated gearbox condition assessment in industrial settings.

The proposed MixNet architecture demonstrates significant potential for enhancing gearbox fault diagnosis in industrial environments. Its high classification accuracy, robustness to noise, and ability to extract discriminative features from spectrogram representations make it a promising solution for real-world applications. Continued research aimed at addressing the current limitations and exploring real-time implementation will further support the development of more sophisticated and reliable predictive maintenance systems for industrial gearboxes, ultimately enhancing operational efficiency, safety, and cost-effectiveness.

Building upon the foundation established in this study, several promising directions for future research can be identified. First, incorporating transfer learning techniques may enable the model to generalize across various types of rotating machinery beyond gearboxes, thereby contributing to the development of more versatile diagnostic systems. Second, exploring self-supervised pre-training methods could enhance feature extraction capabilities, particularly in scenarios with limited labeled data. Third, the integration of attention mechanisms may allow the model to better focus on fault-relevant patterns while filtering out irrelevant information in the spectrogram representations. Fourth, capturing the temporal evolution of faults through recurrent neural networks or temporal convolutional networks may capture the evolutionary behavior of faults over time. Fifth, extending MixNet to support explainable AI techniques would increase model transparency and foster greater trust among maintenance personnel by providing interpretable diagnostic insights. Finally, developing lightweight versions of the architecture suitable for edge computing could enable real-time monitoring on resource-constrained devices deployed directly in industrial environments. These future directions aim to advance the field toward more accurate, versatile, and practically deployable fault diagnosis systems for industrial applications.
